# Magnesium Potentiates the Vortioxetine’s Effects on Physical Performances and Biological Changes in Exercise-Induced Stress in Rats

**DOI:** 10.3390/medicina58101363

**Published:** 2022-09-28

**Authors:** Paula Alina Fotache, Liliana Mititelu-Tartau, Maria Bogdan, Beatrice Rozalina Buca, Liliana Lacramioara Pavel, Ana-Maria Pelin, Andreea-Daniela Meca, Cosmin-Gabriel Tartau, Gratiela Eliza Popa

**Affiliations:** 1 Department of Pharmacology, Clinical Pharmacology and Algesiology, Faculty of Medicine, “Grigore T. Popa” University of Medicine and Pharmacy, 700115 Iasi, Romania; 2 Department of Pharmacology, Faculty of Pharmacy, University of Medicine and Pharmacy of Craiova, 200349 Craiova, Romania; 3 Department of Morphological and Functional Sciences, Faculty of Medicine and Pharmacy, “Dunărea de Jos” University, 800010 Galați, Romania; 4 Department of Pharmaceutical Sciences, Faculty of Medicine and Pharmacy, “Dunărea de Jos” University, 800010 Galați, Romania; 5 Department of Pharmaceutical Technology, Faculty of Pharmacy, “Grigore T. Popa” University of Medicine and Pharmacy, 700115 Iasi, Romania

**Keywords:** vortioxetine, brain-derived neurotrophic factor, BDNF, magnesium chloride, rats, treadmill test

## Abstract

*Background and objectives:* Vortioxetine (VRT) is a relatively new selective serotonin reuptake inhibitor (SSRI) antidepressant and serotonin receptor modulator, approved for the treatment of major depression and generalized anxiety disorder. Depression has been linked with psychomotor disengagement, oxidative stress burden and decreased blood levels of brain-derived neurotrophic factor (BDNF). In our study we performed the experimental investigation of VRT, magnesium and of their association on the rats’ endurance capacity, motor behavior and blood biological disturbances in rats subjected to forced exercise in treadmill test. *Materials and Methods:* The substances were administered orally for 14 consecutive days, as follows: group 1 (control): distilled water 0.3 mL/100 g body; group 2 (Mg): magnesium chloride 200 mg/kg body; group 3 (VRT): VRT 20 mg/kg body; group 4 (VRT+Mg): VRT 20 mg/kg body + magnesium chloride 200 mg/kg body. Magnesium was used as positive control substance with known effects in treadmill test. The consequences of VRT treatment on glucose, cortisol, BDNF and oxidative stress biomarkers (superoxide-dismutase, malondialdehyde, glutathione-peroxidase, lactate dehydrogenase) were also assessed. *Results and conclusions:* The use of VRT resulted in an improvement in motor capacity and an increase of the rats’ endurance to physical effort. The administration of VRT increased the serum BDNF levels and reduced the oxidative stress in rats subjected to physical effort. The association of magnesium potentiated the effects of VRT on physical performances, the antioxidant activity and the decreasing in serum stress markers in treadmill test in rats.

## 1. Introduction

Depression represents a heterogenous multifaceted disorder, as it interferes with disengagement in daily physical activity and alteration of balance between antioxidants and reactive oxygen species (ROS) by increasing oxidative stress [1,2,3]. However, depression is characterized by yet unknown pathogenesis mechanisms [1,2]. Better control of oxidative stress could lead to improved antidepressant treatment outcomes and decreased risk of disease worsening [3]. On the other hand, the beneficial antioxidant role of antidepressants is still under debate as some agents increase concentration of enzymatic oxidative substrates through biogenic amines [3].

Most chemical alterations associated with stress are the result of stimulation of the sympathetic nervous system [4]. At present, many records support the hypothesis that the body’s response to stress is genetically mediated, including above all the effectiveness of the body response to different treatments [5,6,7].

For a long time, it was considered that stress-induced changes in the expression of genes would interfere in the mediation of gene-environmental interaction. Changes in the expression level of some genes, such as those of the brain-derived neurotrophic factor (BDNF), cyclic adenosine monophosphate (cAMP) binding protein, serotonin receptors and some of the components of the hypothalamic-pituitary-adrenal axis (HPA), have been highlighted in various experimental stress models [8].

Recent research results suggest that several mediators, transcription factors and proteins involved in neuronal growth/differentiation also exhibit a modified expression in different experimental models of chronic stress in laboratory animals [9]. Depressive behavior has also been linked with lower plasmatic levels of magnesium (Mg) [10,11]. Chronic administration of Mg proved potential antidepressant activity by acting upon glutamatergic transmission as an N-methyl-D-aspartate receptor antagonist [10]. Pochwat et al. underlined the capacity of Mg to release BDNF and to reduce hyperactivity in rats models, therefore reversing behavioral abnormalities [10]. Selective serotonin reuptake inhibitors (SSRI) such as escitalopram and fluoxetine (widely used antidepressant agents) are also involved in neuroplasticity events via enhancement of BDNF expression which further interferes with upregulation of antioxidant enzymes [12].

Another relatively new antidepressant that exerts neuroprotection and antioxidant activity is vortioxetine (1-[2-(2,4-dimethylphenyl-sulfanyl)-phenyl]-piperazine) (VRT), approved by the European Medicine Agency in 2013 for the treatment of major depression disorder and generalized anxiety disorder [13,14,15]. VRT is currently formulated as immediate release tablets (5 mg, 10 mg, 15 mg and 20 mg) and as oral solution (lactate salt, 20 mg/mL) [13,14,15]. Recent clinical trials have demonstrated its efficacy and safety in treating anxiety in children and teenagers with depressive or anxiety disorder [16]. VRT was classified by the World Health Organization ATC index in the category of “other antidepressants”, group that generally comprises modern antidepressant agents combining different pharmacologic mechanisms, which makes them superior to traditional antidepressants [17,18]. VRT acts as an unique multimodal antidepressant as it simultaneously modulates one or more serotonin receptors and inhibits serotonin reuptake, therefore VRT is considered a modulator and simulator of serotonin [19,20].

Moreover, VRT inhibits the serotonin transporter, but it also displays other pharmacologic effects in the brain: it acts as an agonist of serotonin receptors 5-HT_1A_ (full agonist), 5-HT_1B_ (partial agonist) and an antagonist of the receptors 5-HT_1D_, 5-HT_3_ and 5-HT_7_ [21]. Preclinical studies have shown differences in receptor occupancy in laboratory animals compared to humans: thus, VRT has a lower affinity for the 5-HT_7_ and 5-HT_1A_ receptors in rats than in humans [22,23,24].

In rats, the inhibition of serotonin transporter and of 5-HT_3_ are evident at doses of 0.3 up to 10 mg/kg), whereas the other 5-HT receptors are occupied in higher doses. Also, animal studies have revealed that VRT influences other neurotransmitter systems, such as: norepinephrine, dopamine, histamine and acetylcholine in various regions of the brain, mostly because of the modulation of 5-HT receptor system [22,25,26]. These actions may explain the positive influence of vortioxetine on cognitive function, demonstrated in animal experiments and clinical trials. 

In humans, VRT has a bioavailability of cca 75% and a mean elimination half-life of cca 60 h, reaching its steady state in 12 to 14 days, and its pharmacologic actions are attributed to the parent drug, since its metabolization in the body leads to inactive metabolites. VRT does not influence the P450 enzymes by induction or inhibition, so it is more advantageous than other antidepressants (e.g., fluoxetine or paroxetine) in combination with other drugs, because of low risk of interactions [24,27].

However, few information is available regarding antioxidant and neuroprotective activity of VRT, therefore the aim of the present study was the experimental investigation of the effects of VRT and Mg on the ability of laboratory animals (rats) to withstand physical exertion and motor behavior. We also intended to evaluate VRT and Mg influence on BDNF, glucose and cortisol serum levels as well as upon oxidative stress biomarkers [such as superoxide-dismutase (SOD), malondialdehyde (MDA), glutathione-peroxidase (GPx) and lactate dehydrogenase (LDH)]. To the best of our knowledge, this is the first study that investigates the effects of VRT and Mg on physical performance and biological changes in experimental animals subjected to stress through forced exercise.

## 2. Materials and Methods

### 2.1. Laboratory Animals

Thirty healthy male rats, white Wistar breed, lacking pathogenic conditions, not genetically modified (weighing between 150–200 g, 6–7 weeks old), were used for the experimental research. The animals were procured through the biobase of the University of Medicine and Pharmacy ‘Grigore T. Popa’ Iaşi and were supplied by the ‘Cantacuzino’ National Medical-Military Institute for Research and Development, Băneasa Resort, Bucharest, Romania.

Single-sex rats were used, taking into consideration the literature data concerning the differences between animal sexes in terms of reactivity on experimental patterns of behavior and differences in pharmacodynamic effect of different antidepressants [28].

The rats were brought to the experiment room the day before for accommodation, being housed in special cages (for two animals each) and were kept in standard laboratory conditions with a constant temperature of 21 °C ± 2 °C, 50–70% RH and with an alternating lighting cycle (light/dark ratio of 12/12 h). In order to prevent chronobiological influences, the investigations were carried out in the same time interval (between 8–12 a.m.).

The animals received drinking water *ad libitum* provided by means of special drinkers and standardized granular feed (pellets), except for the days when the experiments took place. The amount of pellets consumed by each animal, as well as their weight, were measured at the beginning of each experiment day. On the day of the experiment, half an hour before the start of the investigations and for 3–4 h during the experiments, the animals did not receive drinking water, as they would have consumed an uneven volume of fluid. This could have changed the distribution volume of the administered drugs and consequently their pharmacokinetics, accelerating their clearance from the body and decreasing their pharmacodynamic effect.

The presence of food in the gastric cavity might influence differently drug pharmacokinetics, even within the same group, with differences in the percentage of absorption and consequently in blood concentration, depending on the presence or absence of food in the stomach. From a procedural point of view, if the consumption of water and food was allowed, then from the very beginning of the experiment, a difference in the pharmacodynamic effects between animal groups would be noticed.

### 2.2. Substances

VRT (vortioxetine hydrobromide, catalogue name SML3388, ≥98%) and magnesium chloride (Mg) (catalogue name M8266, anhydrous, ≥98%) were obtained from Sigma Chemical Co., Steinheim, Germany. Distilled water was purchased from Zentiva Pharmaceutical Company Romania. The substances were dissolved in distilled water, the solutions being prepared extemporaneously.

In order to achieve similar experimental conditions for all the animals tested, distilled water-the solvent for the substances under study, was administered only to the control group, by gavage (using a flexible silicone eso-gastric probe), which provided safety that the animals received the same volume of liquid (hence the same vehicle), which was the positive control in the experiment.

### 2.3. Experimental Protocol 

The researches took place within CEMEX (Advanced Center for Research and Development in Experimental Medicine) of University of Medicine and Pharmacy ‚Grigore T. Popa’ from Iasi. The researches carried out followed the approval by the Ethics Commission of University of Medicine and Pharmacy ‚Grigore T. Popa’ from Iaşi (Certificate No. 25/14.07.2020), in strict accordance with the International Ethical Regulations Regarding Animal Experiments. 

#### 2.3.1. The Treadmill Test

For the experiment, 4 groups of 6 animals each were used, which received the substances orally (using an esogastric tube), in a single daily dose for 14 consecutive days, as follows:

Group 1 (Positive control): distilled water 0.3 mL/100g body;
Group 2 (Mg): magnesium chloride 200 mg/kg body [29,30];
Group 3 (VRT): VRT 20 mg/kg body [31];
Group 4 (VRT+Mg): VRT 20 mg/kg body + magnesium chloride 200 mg/kg body.

The fifth group (negative control) consisting in 6 rats treated with distilled water, not subjected to treadmill test, was used for the comparative analysis in order to identify the changes induced by forced stress on the serum level of some parameters.

During the experiment the normal behavior of laboratory animals was observed, noting the general condition, appearance of hair, respiration, gait, reaction of the animal to various stimuli. All behavioral disturbances, changes in posture or handling reactions, as well as the appearance of clonic or tonic movements, stereotyped behaviors (e.g., excessive cleansing, repeated circular movements), and any other behavior were also noted, as well as other bizarre behavior (self-mutilation, going backwards).

In order to investigate the effects of the studied antidepressant on the capacity of physical effort, physical endurance and locomotor activity, the test of the treadmill was used, which was performed in perfect silence, to avoid stressing the animal. On the 14th day of the experiment after the administration of the substances, the animals were left to rest for 15 min, after which they were subjected to the forced effort test.

At the beginning of the experiment, the animal is placed in the area of the grid, at the end of the constantly rotating belt, and is allowed to move continuously. It will run in the opposite direction to the one in which the belt rotates. For each animal, the experimentation session lasted 10 min. The parameters within which the experiment was carried out were established: the speed of the belt (10 m/min), the tilting angle of the travel slope (at 5 degrees) and the intensity of the shocks administered to the animal (1.2 mA, 3 Hz current).

During the laboratory accommodation period, the rats underwent a training session until they were able to walk continuously on the treadmill without the assistance of the experimenter.

For each experiment session, the following parameters were monitored: total distance travelled, number of electric shocks applied, moment of application of electric shocks, latency of the animal resistance until the first electrical impulse was applied, number of touches of the opposite end of the conveyor belt. It is considered that the number of interruptions of continuous running on the belt corresponds to the number of electrical impulses applied to the animal, in order to force it to continue moving if it tries to stop or it falls [32,33]. 

In the treadmill test, for each individual experiment session, it is considered that the decrease of the time duration for the application of electric shocks or the reduction of the number of electrical impulses applied to the animal for forcing the continuation of movement, means an effect of increasing the resistance to effort, produced by the tested substance [34].

On the contrary, the prolongation of the time for the application of electric shocks or the increase of the number of electric shocks, necessary to boost the motor activity of the animal are an expression of decrease of resistance to physical effort, produced by the investigated substance [32]. The increase of the total distance travelled by the animal by running on the treadmill corresponds to an increase the resistance to physical effort and a stimulation of the motor activity, achieved by the investigated substance [35].

On the other hand, the decrease of the total distance of animal movement on the treadmill is considered to be linked to a decrease in the resistance to physical effort, with a decrease of motor activity, determined by the tested substance [32].

#### 2.3.2. The Laboratory Investigations

At 14 days in the experiment, after the tested groups were subjected to forced exercise, 0.3 mL of blood was collected from the animal lateral tail vein for laboratory analysis. The biochemical and immunological laboratory determinations were performed using special analyzers for each test or spectrophotometry. 

This blood collection technique is less stressful, does not require anesthesia, allows multiple blood collections from a single animal and provides a relatively large sample volume so that multiple tests can be performed on a single sample [36,37].

The blood glucose levels were determined using appropriate tests and kits (VITROS 750 XRC, using Johnson & Johnson reagents for biochemical tests, and specific HEMA-VET device for hematological investigations). Cortisol dosing (basal or in combination with stress tests) provides information on adrenal, pituitary and hypothalamic function. Serum hormone levels were determined using the electrochemiluminescence (ECLIA) immunochemical method. The determination of the serum level of BDNF was performed by ELISA method (using specific kits) [38,39].

SOD activity analysis was determined spectrophotometrically (using a Shimadzu 1800 Spectrophotometer, Kyoto, Japan, using RANSOD specific kit (catalogue name SD125) from RANDOX Laboratories Ltd., Warsaw, Poland) on blood samples (0.3 mL) collected on ethylenediaminetetraacetic acid (EDTA), method based on inhibition of the reduction of nitroblue tetrazole with xanthine-xanthine oxidase, used as a superoxide generator [40].

Estimation of MDA activity in blood was performed by high performance liquid chromatography (HPLC) with fluorescence detection on blood samples (0.3 mL) collected in vacuum cleaners with EDTA using specific kit (catalogue name ABIN772058) from Redox, Bucharest, Romania.

Measurement of serum GPX activity was performed spectrophotometrically by the dithio-nitrobenzoic acid reagent (DTNB) method on blood samples (0.3 mL) collected in EDTA vacutainers using a specific RANSEL kit (catalogue name RS504) from RANDOX Laboratories Ltd., Warsaw, Poland.

The protocol that accompanied the reagents used for each kit was followed.

### 2.4. Statistical Data Processing

The obtained data were expressed as the arithmetic mean ± standard deviation (S.D.) of the mean values and statistically analyzed using the SPSS 19.0 software for Windows 10 using the ANOVA one-way method. Thus, it was possible to estimate the significance of the differences obtained within the same group of laboratory animals and the differences between the tested groups compared to the control group. In the statistical analysis of the data, the values of the *p* (probability) coefficient less than 0.05 were estimated as statistically significant, compared to the control group.

## 3. Results

### 3.1. The Treadmill Test

The forced locomotion test allows the exploration of spontaneous motor activity, motor coordination and the endurance capacity of the laboratory animal, which is placed on the moving test strip. When the rat gets tired and stops at the grid, electric shocks are applied in the form of impulses, which will act as a stimulus, motivating the animal to continue moving on the belt.

With the help of this exercise test, the distance traveled by the laboratory animal while moving on the treadmill was measured, in order to assess the resistance capacity to physical effort, in the period of time given to the determinations.

**Figure 1:**
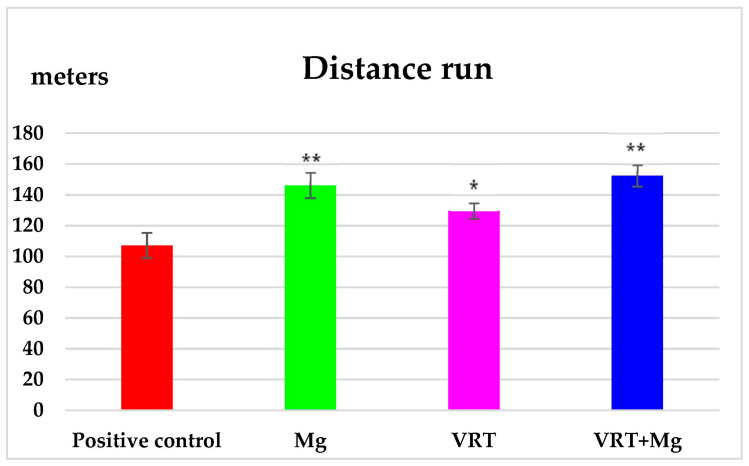
The effects of Mg, VRT and VRT+Mg on the distance run in the treadmill test in rats. Each point represents the mean ± S.D. of the average of distance run, for 6 animals per group. The values of the coefficient * *p* < 0.05, ** *p* < 0.01 were considered statistically significant versus positive control.

The statistical analysis of the data obtained from the treadmill test highlights that within 10 min of the experiment, Mg increased the distance traveled by the animal on the belt (146.17 ± 8.26 m), statistically significant (** *p* < 0.01) compared to the positive control group (107.17 ± 8.16 m) in the forced locomotion test. The administration of VRT for 14 days was associated with the significant (* *p* < 0.05) lengthening of the distance traveled by the animal on the treadmill (129.50 ± 5.13 m), compared to the group that received distilled water on the exercise test (Figure 1). The addition of Mg, substantially extended the run distance (152.33 ± 7.03), much more pronounced than measured for the VRT, respectively Mg group (Figure 1).

The forced locomotion test can determine how many times the rat reaches the opposite end of the strip, an element that is directly correlated with its resistance to stress. Logically, the increase in the number of touches of the opposite end of the treadmill compared to the control, corresponds to an increase in motor capacity and endurance of the animal, which overcomes fatigue and manages to cover the entire length of the treadmill. Otherwise, the decrease in the number of touches of the opposite end of the strip compared to the control, is due to the decrease of the motor capacities, and of the resistance of the rat to physical effort.

**Figure 2:**
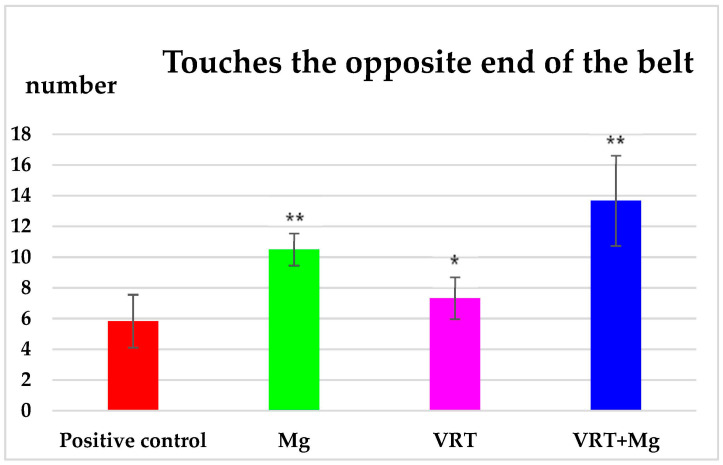
The effects of Mg, VRT and VRT+Mg on the number of touches of the opposite end of the belt in the treadmill test in rats. Each point represents the mean ± S.D. of the average of the touches number, for 6 animals per group. The values of the coefficient * *p* < 0.05, ** *p* < 0.01 were considered statistically significant versus positive control.

The statistical processing of the results obtained from the treadmill test highlighted that the oral administration of Mg resulted in an increase in the number of touches of the opposite end of the conveyor belt (10.50 ± 1.05), statistically significant (** *p* < 0.01) compared to the positive control group (5.83 ± 1.72). The use of VRT for 2 weeks was accompanied by a significant (* *p* < 0.05) increase in the number of touches of the opposite end of the conveyor belt (7.33 ± 1.37) compared to distilled water group, at forced locomotion test (Figure 2). The association of VRT with Mg resulted in a considerable increase in the number of touches of the opposite end of the device (13.67 ± 2.94), comparing with both VRT and Mg groups (Figure 2).

In the event the motor activity of the animal decreases or it stops in the area of the grid during the effort, it is necessary to apply electric shocks to stimulate resistance to effort and boost the continuation of running on the treadmill.

With the help of the forced locomotion test, the time can be determined until the application of the first electric shock, a parameter that allows the appreciation of the time of the animal physical resistance to effort, until its impulse is necessary to continue running on the treadmill.

**Figure 3:**
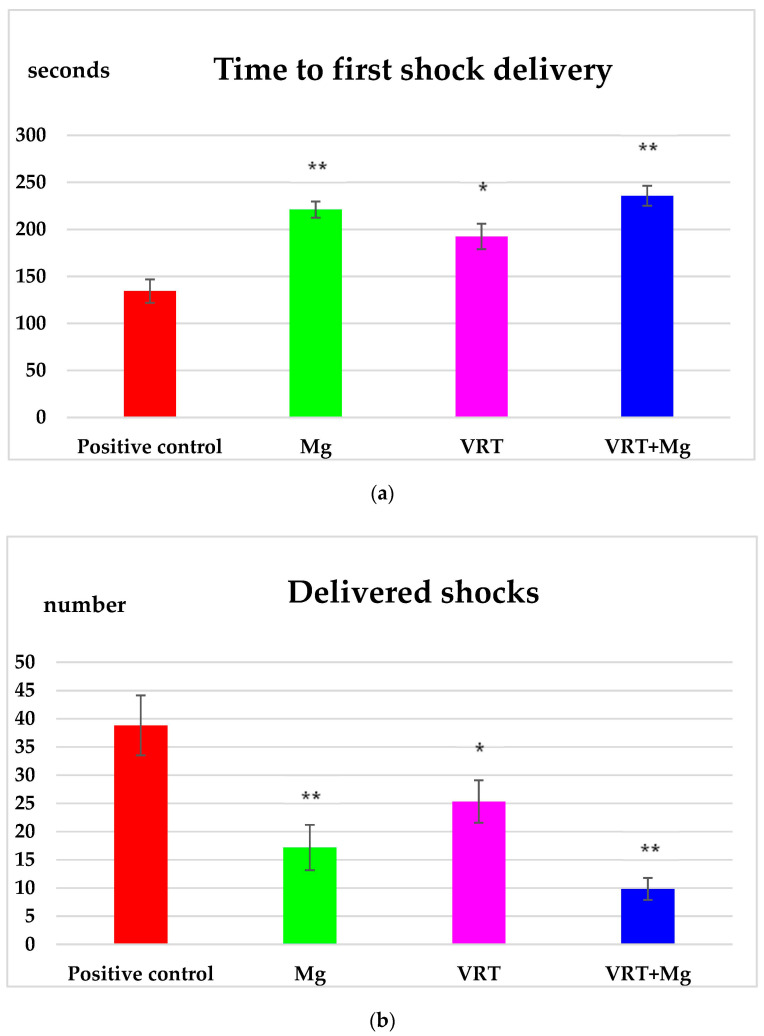

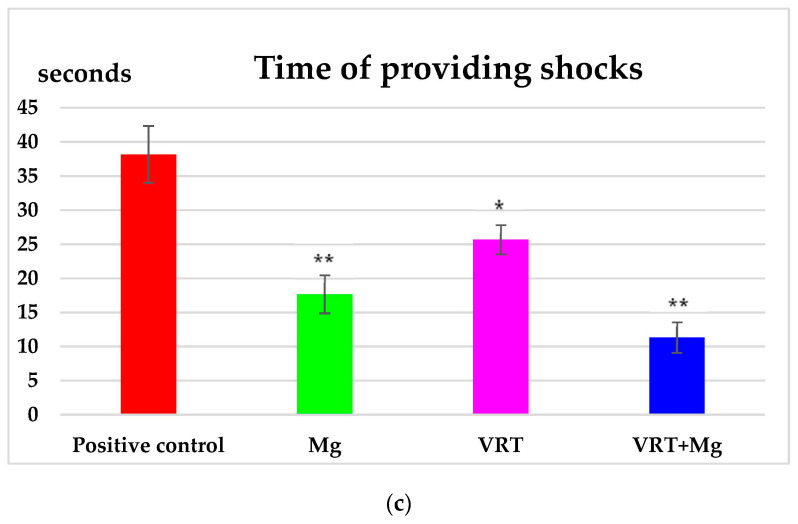
The effects of Mg, VRT and VRT+Mg on time to first shock delivery (**a**) the number of delivered shocks (**b**) and time of providing shocks (**c**) in the treadmill test in rats. Each point represents the mean ± S.D. of the average values for 6 animals per group. The values of the coefficient * *p* < 0.05, ** *p* < 0.01 were considered statistically significant versus positive control.

The centralization of the data recorded at the test of the conveyor belt highlights the fact that the treatment with Mg determined an important (** *p* < 0.01) prolongation of the time duration until the application of the first electric shock (221.00 ± 8.65), compared to the positive control rats (134.33 ± 12.48). The administration of VRT was accompanied by a notable (* *p* < 0.05) increase in the latency until the application of the first electric shock (192.50 ± 13.49), compared to the distilled water group. These effects of VRT+Mg were more intense compared to those of the single use of VRT, respectively of Mg on the time to first shock delivery (215.17 ± 4.49), over the same session of the evaluation (Figure 3a). By means of the treadmill test, counting the number of shocks applied to the animal under conditions of forced effort serves to assess the need to stimulate the motor activity if the animal gets tired. 

Monitoring the behavior of the animal on the forced locomotion test showed that the use of Mg, produced a marked (** *p* < 0.01) decrease in the number of electric shocks applied to the animal (17.17 ± 4.02), compared to the positive control group (38.83 ± 5.31) at the exercise test. The treatment with VRT for14 days resulted in a lower need for electric stimulation (15.14 ± 3.72), statistically significant (* *p* < 0.05) compared to the group that received distilled water in this behavioral test. The combination of VRT with Mg diminished the number of provided shocks (9.83 ± 1.94), more accentuated than VRT group, as well as than Mg group (Figure 3b).

The study of the data counted in the conveyor belt test revealed that administration of Mg was correlated with a significant reduction in the total time of application of electric shocks (17.67 ± 2.80), statistically significant (** *p* < 0.01) compared to the positive control group (38.17 ± 4.17) in the forced locomotion test. The use of VRT was linked with a noteworthy (* *p* < 0.05) decreased the total time for the application of electric shocks (25.67 ± 2.16), statistically significant (* *p* < 0.05), compared to the group treated with distilled water in this behavioral model. The treatment with VRT+Mg led to a more important diminution of the time period for providing the electric impulses (11.33 ± 2.25) than counted for VRT, respectively Mg groups (Figure 3c).

### 3.2. The Laboratory Investigations

A significant (♦♦ *p* < 0.01) increase in serum glucose values (116.33 ± 1.53) was highlighted in rats subjected to forced physical effort compared to negative control animals (88.50 ± 6.24) (Table 1). Mg treatment was associated with a marked (*p < 0.05) reduction in blood sugar compared to the group that received distilled water on the exercise test. The administration of VRT caused an important decrease (* *p* < 0.05) in glycemia compared to the positive control group. Laboratory analysis of blood samples from VRT+Mg group showed a more accentuated reduction in glucose levels in comparison with both the VRT group and the Mg group (Table 1).

In the animals subjected to the forced locomotion test, a substantial (♦ *p* < 0.05) increase in the serum cortisol level (0.19 ± 0.02) was found compared to negative control group (0.14 ± 0.01). No significant changes in blood cortisol values were noted between the groups of animals receiving Mg, VRT and positive control rats (Table 1). The association of VRT with Mg diminished the serum cortisol levels (0.15 ± 0.02) statistically significant (* *p* < 0.05) versus positive control group (Table 1).

The physical effort induced a substantial (♦♦ *p* < 0.01) diminution in blood levels of BDNF (477.50 ± 33.23), compared to negative control group without effort (575.67 ± 47.25). The use of Mg (569.50 ± 31.82) and VRT (563.00 ± 41.01) resulted in an important increase in BDNF values, statistically significant (** *p* < 0.01) comparing with positive control group, the effects of VRT being less intese than those of Mg in the experiment. The increase in the serum level of BDNF in the group treated with the VRT+Mg combination was much more pronounced than that observed for the groups that received the substances separately (Table 1).

In rats subjected to forced physical effort were observed relevant decrease (♦♦ *p* < 0.01) in SOD (15.00 ± 2.83), GPx (194.00 ± 35.36) and LDH (2407.50 ± 21.92) activity, and a reduction in blood level of MDA (38.5 ± 2.40) compared to rats without effort (Table 2).

The use of Mg was accompanied by a marked (** *p* < 0.01) increase of SOD (23.00 ± 1.41), GPX (311.50 ± 113.84) and LDH (2094.00 ± 156.98) values, respectivelly a reduction of blood MDA (26.65 ± 3.46) levels compared to the group that received distilled water under stress conditions (Table 2). 

VRT treatment resulted in an obvious intensification in SOD (22.33 ± 2.12), GPx (309.00 ± 165.46) and LDH (2100.50 ± 144.96) activity, and a decrease of serum MDA (26.93 ± 1.12) values, versus the positive control group in the treadmill test. Much more intense effects of increasing the levels of the evaluated antioxidant enzymes, but also the significant reduction of the activity of the pro-oxidant enzyme were found for the VRT+Mg group, compared to the groups treated only with VRT, respectively Mg alone (Table 2).

**Figure 4:**
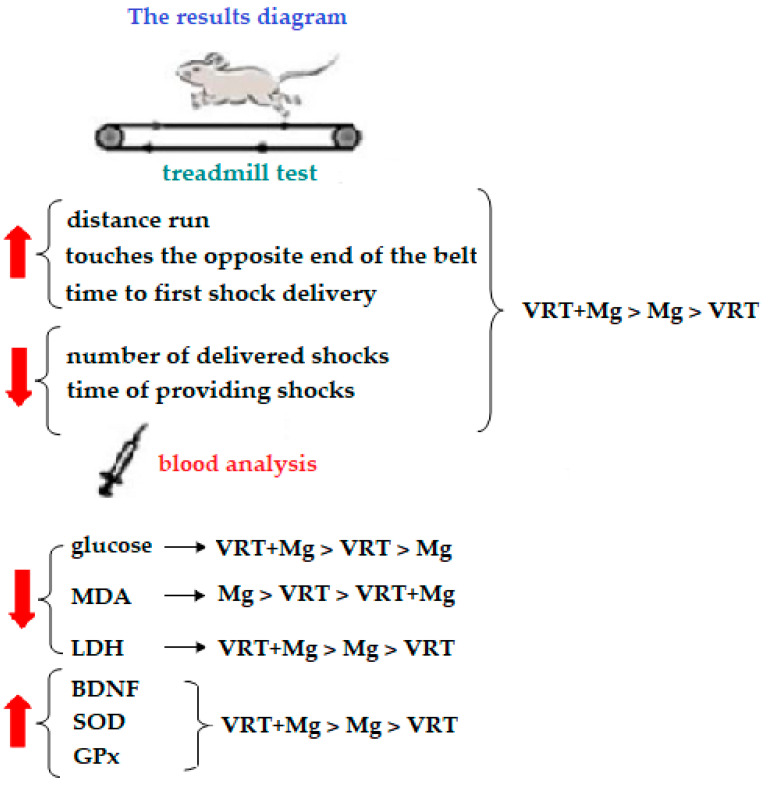
The main findings of the study.

The results of the study are summarized in Figure 4.

## 4. Discussion

Clinical experience has shown that stressful situations increase the prevalence of a wide range of health disorders, including coronary artery disease, chronic lung disease and certain type of cancers. Stress can also have negative consequences for behavioural adaptations and predisposal of certain people to depressive disorders or anxiety. However, there is a strong inter-individual variability in susceptibility to stress, so that most people are generally resistant, can maintain their physiological and mental functions within normal limits, despite the fact that that they are undergoing stress, sometimes of high intensity [41,42].

BDNF is a protein found in the brain and spinal cord which helps the neurons to survive by playing a role in growth, maturation, and maintenance of these cells. In the brain, the BDNF protein is active at the synapses site. The synapses can change and adapt over time in response to experience, a characteristics called synaptic plasticity. Animal studies have demonstrated that stress reduces BDNF expression or activity in the hippocampus and this reduction can be prevented by treatment with antidepressant drugs. BDNF levels can therefore be useful markers for clinical response or improvement of depressive symptoms due to stress factors [12,43].

It is suggested that the physical activity parameters, such as the type of effort, intensity, effort duration, are important elements that influence the behavioral and affective response of the laboratory animal [44,45].

Experimental research has revealed that physical exercise of reduced or moderate intensity produces anxiolytic and antidepressant effects in rodents, while acute physical exercise of high intensity is associated with anxious and/or depressive-type behavior [45,46,47].

The reduced serotonin level in the brain is the essential element in the emergence of anxiety and depression, which explains the favorable influence of serotonin reuptake inhibitors in mediating the pathogenic pathways involved, with the improvement of these pathological conditions [44].

In our research, we studied the influence of VRT administration on exercise capacity and motor behavior in rats, compared to the use of magnesium chloride, whose facilitating effects on exercise resistance are known in the treadmill test [48].

In parallel, we studied the changes produced by this antidepressant on some parameters in the blood and on some markers of the oxidative processes, in rats subjected to stress by forced exercise.

The effects of VRT were found to be inferior compared to Mg under the same experimental conditions on the exercise test. In VRT group, it was observed a noticeable decrease in blood glucose levels compared to the positive control group, effects which were more intense than of Mg. Neither Mg, nor VRT were shown to significantly influence the serum cortisol levels during the experiment. The use of Mg and VRT attenuated the oxidative stress in animals subjected to forced exercise, the effects of VRT being weaker than those produced by Mg.

Our results are consistent with a number of existing literature communications regarding the influence of various antidepressants on the serum BDNF values and on stress biological and behavioural manifestations in various experimental models in laboratory animals [43,49,50].

One of the currently circulating assumptions is that the decrease in BDNFexpression and possibly in other growth factors contributes to the occurrence and manifestation of depression. Furthermore, it was demonstrated that the up-regulation of BDNF plays a key role in response to antidepressant treatment [9,51,52,53]. Clinical trials have pointed out that patients with major depressive disorders have low levels of BDNF in their blood, which is considered one of the major neurotrophic factors, important for the synaptic plasticity [54,55]. Low levels of BDNF, as well as of other neurotrophic factors, could contribute to the atrophy of certain limbic structures, including the hippocampus and prefrontal cortex, which has been observed in depressed subjects [56]. Instead, neurotrophic actions of antidepressants can reverse the neuronal atrophy and cell loss, which can explain the therapeutic efficacy of these drugs [57].

Classical antidepressants, such as the selective serotonin reuptake inhibitors, selective norepinephrine reuptake inhibitors and monoamine oxidase inhibitors, therapeutic agents that interfere the modulation of brain monoamine levels, have limited efficacy and a delayed therapeutic response of several weeks or months. These typical antidepressants have been shown to increase the expression mRNA (messenger ribonucleic acid) of BDNF, with a delay of weeks [58]. Other investigations have shown that the treatment with paroxetine in depressive patients reduces DNA methylation and increases the expression of BDNF [59,60]. 

Other therapeutic methods administered to patients with major depressive disease, such as electroconvulsive shock treatment, rapidly increase the BDNF levels in the prefrontal and hippocampus cortex, much more higher values being noted in chronic treatment [61].

In this study we demonstrated for the first time that VRT, an antidepressant with multimodal action, improves endurance, balances the stress-induced disturbances of biochemical parameters, reduces oxidative stress, preventing the development of characteristic depressive behavior by lowering serum levels of BDNF.The limitations of our study are represented by the small number of animals and the reduced period of time for VRT administration, but the results encourage us to continue the research in this direction. We also intend to compare the effects of VRT to those of other antidepressants with a complex mechanism of action.

## 5. Conclusions

In our experiment, VRT induced an improvement in motor skills and resistance to physical effort in the treadmill test. The association of VRT with Mg led to an improvement of the endurance capacity of rats subjected to forced effort. The administration of VRT reduced the blood levels of some specific stress markers, and increased BDNF levels. The addition of Mg was correlated with an enhancing of VRT`s antioxidant activity and a decreasing in stress-induced biochemical disturbances in treadmill test in rats.

Additional studies on a larger number of animals and with the histopathological analysis of the liver and kidneys should provide important perspectives for future clinical studies and medical applicability.

## Figures and Tables

**Table 1:** The effects of VRT, Mg and VRT+Mg on the blood level of glucose, cortisol and BDNF in treadmill test in rats. Each point represents the mean ± S.D. of the average values for 6 animals per group. The values of the coefficient ♦♦ *p* < 0.01, ♦ *p* < 0.05 were considered statistically significant versus negative control; ** *p* < 0.01, * *p* < 0.05 were considered statistically significant versus positive control.

Group	Glucose (mg/dL)	Cortisol (pg/mL)	BDNF (pg/mL)
Negative control	88.50 ± 6.24	0.14 ± 0.01	575.67 ± 47.25
Positive control	116.33 ± 1.53 ♦♦	0.19 ± 0.02 ♦	477.50 ± 33.23 ♦♦
Mg	93.33 ± 8.02 *	0.16 ± 0.03	569.50 ± 31.82 **
VRT	92.00 ± 1.41 *	0.16 ± 0.05	563.00 ± 41.01 **
VRT+Mg	89.17 ± 3.25 **	0.15 ± 0.02 *	573.33 ± 45.78 **

**Table 2:** The effects of VRT, Mg and VRT+Mg on the activity of SOD, MDA, GPx and LDH in treadmill test in rats. Each point represents the mean ± S.D. of the average values, for 6 animals per group. The values of the coefficient ♦♦ *p* < 0.01 were considered statistically significant versus negative control; ** *p* < 0.01, * *p* < 0.05 were considered statistically significant versus positive control.

Group	SOD (μg/mL)	MDA (nmol/L)	GPx (pg/mL)	LDH (U/L)
Negative control	24.33 ± 2.46	27.83 ± 3.16	321.17 ± 33.27	2069.67 ± 21.92
Positive control	15.00 ± 2.83 ♦♦	38.5 ± 2.40 *	194.00 ± 35.36 ♦♦	2407.50 ± 21.92 ♦♦
Mg	23.00 ± 1.41 **	26.65 ± 3.46 **	311.50 ± 113.84 **	2094.00 ± 156.98 **
VRT	22.33 ± 2.12 *	26.93 ± 1.12 *	309.00 ± 165.46 **	2100.50 ± 144.96 *
VRT+Mg	24.17 ± 2.33 **	27.65 ± 2.59 **	319.50 ± 101.41 **	2075.33 ± 132.83 **

## Data Availability

Not applicable.
